# A pilot add-on Randomized-Controlled Trial evaluating the effect of binaural beats on study performance, mind-wandering, and core symptoms of adult ADHD patients

**DOI:** 10.1192/j.eurpsy.2022.701

**Published:** 2022-09-01

**Authors:** F. Malandrone, M. Spadotto, M. Boero, I.F. Bracco, F. Oliva

**Affiliations:** 1 University of Turin, Clinical And Biological Sciences Department, Orbassano, Italy; 2 University of Turin, Department Of Clinical And Biological Sciences, Orbassano (TO), Italy; 3 University of Turin, Departiment Of Neuroscience “rita Levi Montalcini”, Orbassano, Italy

**Keywords:** adhd, Binaural beats, Non-pharmachological intervention, Brain stimulation

## Abstract

**Introduction:**

ADHD is a neurodevelopmental disorder that frequently persists throughout adulthood. Binaural Beats (BB) are auditory perceptions occurring when two soundwaves of slightly different frequency are carried separately to the ears; they might modulate brain activity and performance.

**Objectives:**

To evaluate BB efficacy on studing performance, mind-wandering, and core symptoms of a sample of adult ADHD outpatients

**Methods:**

In this randomized-controlled trial we recruited a sample of University students in pharmacological treatment for adult ADHD. A track with 15 Hz BB (415 Hz to right and 400 Hz to the left ear) was delivered to the intervention group; whereas, a placebo track consisting of two identical frequencies (400 Hz) was administered to control group. The RCT consisted of a baseline assessment (T0) and two fortnightly follow-ups (T1-T2). Each time, the patient filled the ADHD-RS-5 (ADHD Rating Scale-5) and the MEWS (Mind Excessively Wandering Scale) and executed an online version of SART (Sustained Attention to Response Task). The effect of self-administered acoustic stimulation during individual studying sessions was estimated by a subjective studying performance (SSP) evaluation questionnaire.

**Results:**

A significant improvement of SSP from baseline assessment (T1) to the last observation (T3) was detected in BB group only (mean differences= 2.7, p<.001). A significant between-group contrast for SSP was also found at T3. No other significant changes were detected for ADHD-RS, MEWS and SART at p<.05 level.

**Conclusions:**

BB seem to improve subjective studying performance and ADHD symptoms severity. These preliminary findings must be confirmed in larger sample.

**Disclosure:**

No significant relationships.

